# Supplementary material for: Rhizosphere Fungal Dynamics in Sugarcane during Different Growth Stages
Source: Int J Mol Sci. 2023 Mar 16;24(6):5701. doi: 10.3390/ijms24065701 (PMC10052501; doi:10.3390/ijms24065701)
Supplement: Supplementary file 1 [file ijms-24-05701-s001.zip › ijms-2226913-supplementary.pdf]

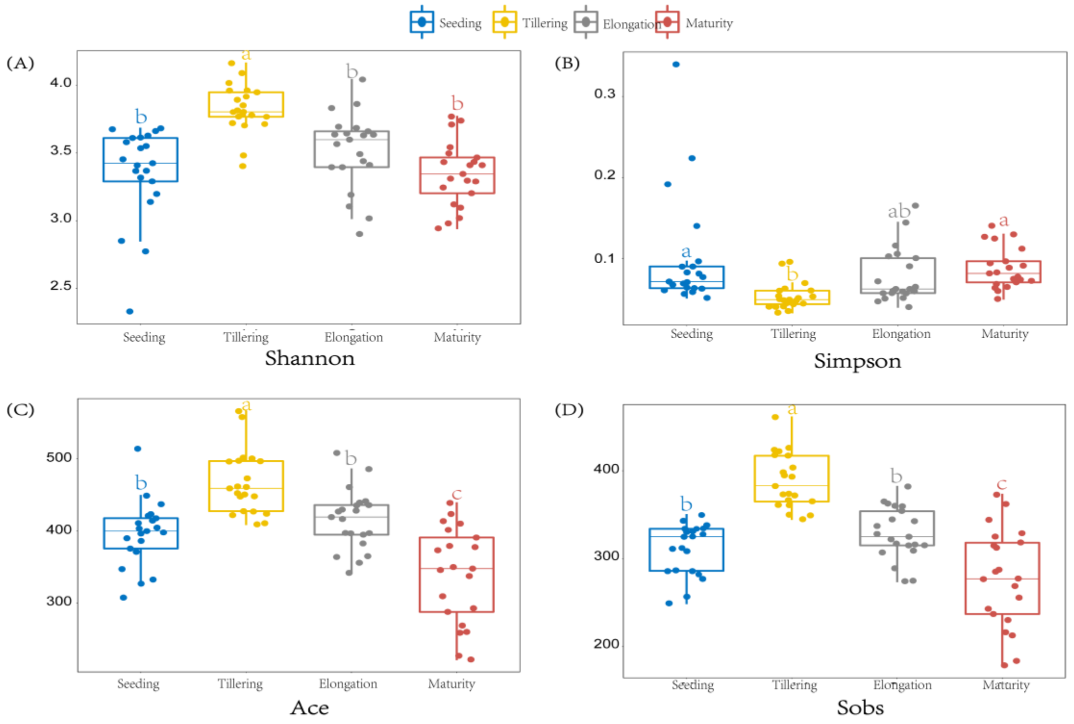

Figure S1. Box plots of rhizosphere microbial α-diversity of sugarcane in different seasons. The horizontal bars in the boxes represent the median. All samples are plotted as individual points. Letters "a, b, c" represent significant differences between groups (FDR adjusted $p < 0.05$).

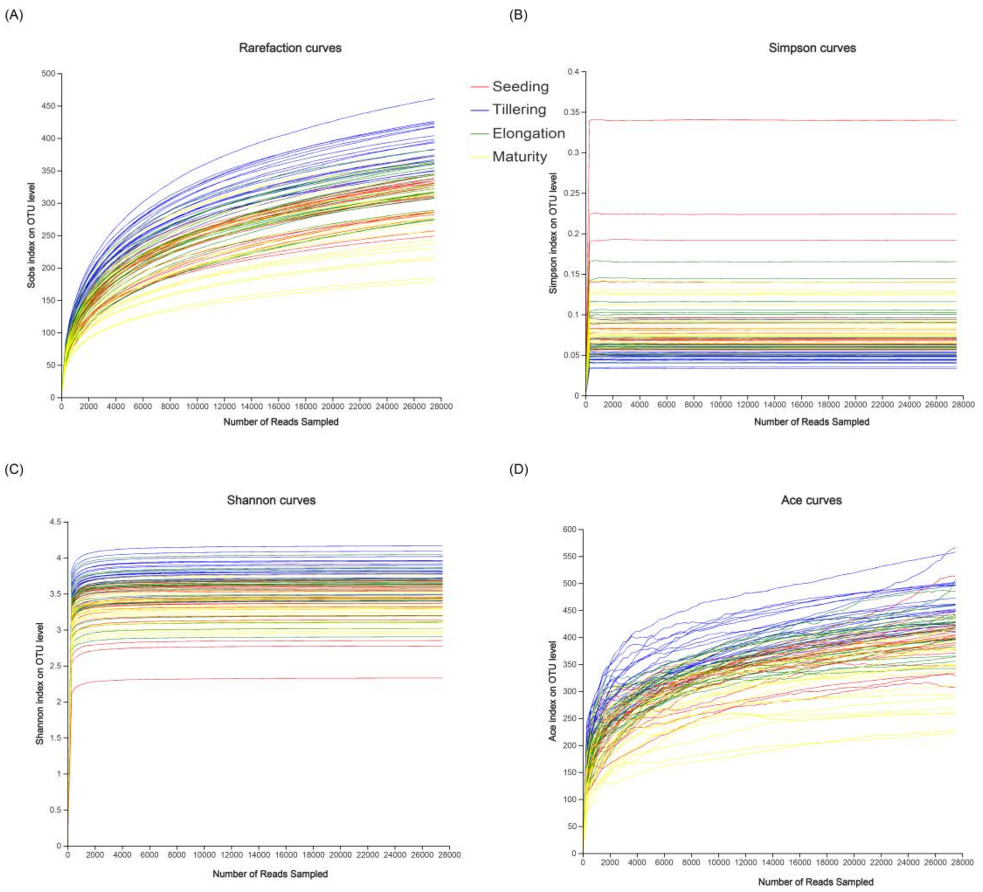

Figure S2. The rarefection curve of fungal alpha diversity index, the horizontal coordinate is the number of reads and the vertical coordinate is the different α-diversity indices. Different colors in the picture represent different growth stages.

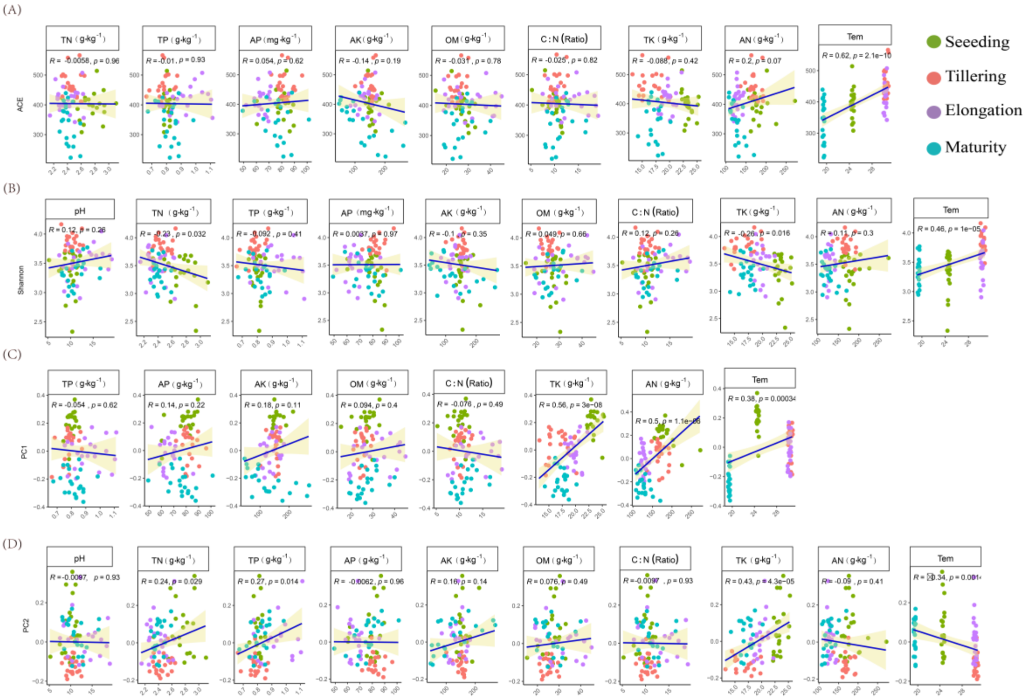

Figure S3. Linear relationships among selected microbial characteristics and soil physicochemical variables ($p < 0.05$). The ordinates of each row are the same, and the abscissas are soil properties. Different colors indicate different growth stages.

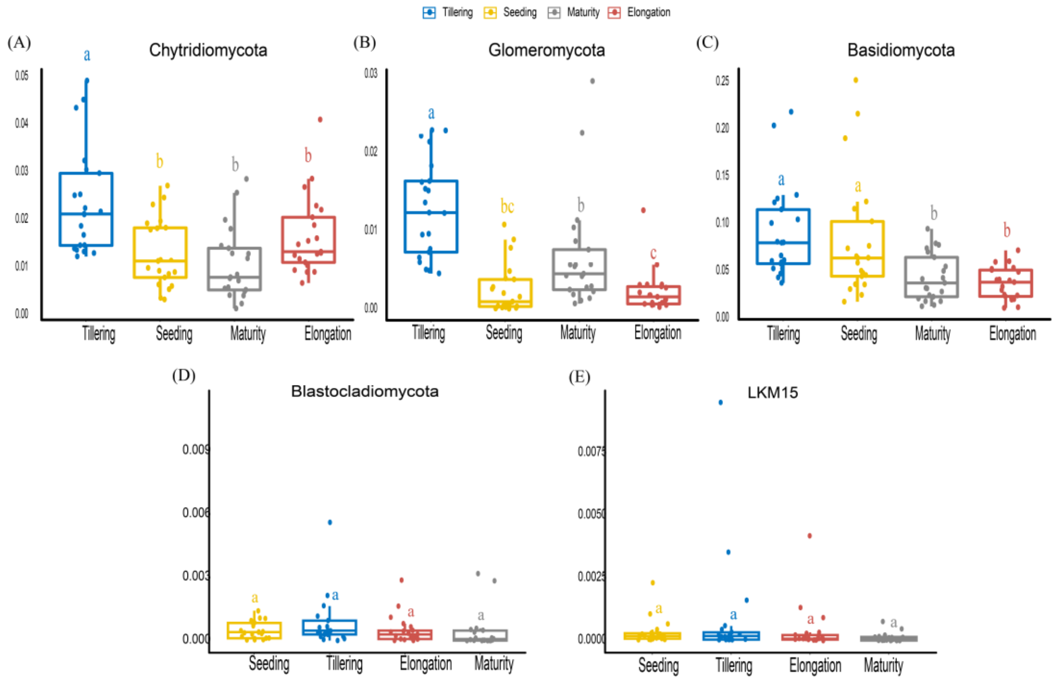

Figure S4. The box plot shows the changes of rhizosphere fungi in four

different sugarcane growth stages. Different letters indicate significantly different groups (FDR adjusted $p < 0.05$). Different colors represent different growth stages. The horizontal bars within boxes represent medians.

Table S1. Summary table of fungal genera declining or enriched in sugarcane roots in the comparison between seasons.

	Order of comparison		
	**Seeding-Tillering**	**Tillering-Elongation**	**Elongation-Maturity**
**Depleted**	Tremellales	Chytridiomycota	Eurotiales
	Eurotiales	Glomeromycota	Sordariales
	Hypocreales	Saccharomycetales	Chytridiomycota
	Saccharomycetales	Pezizales	Hypocreales
	Ascobolaceae	Salpingoeca	Ascomycota
	Pleosporales	Ascomycota	Sordariomycetes
	Dothideomycetes	Agaricomycetes	Pleosporales
	Ascomycota	Galactomyces	Dothideomycetes
	Fusarium	Eurotiales	Saccharomycetales
	Sordariomycetes	Hypocreales	Basidiomycota
	Boeremia	Tremellales	Tremellales
	Tilletiaria	Microbotryomycetes	Euglyphida
	Capnodiales	Dissoconium	Colpodidium
	Trichosporon	Hydnodontaceae	Capnodiales
	Tremella	Cryptococcus	Leotiomycetes
	Thysanophora	Orbiliales	Agaricomycetes
	Chytridiomycota	Monosiga	Aleuria
	Exobasidiomycetes	Tilletiaria	Hydnodontaceae
	Leotiomycetes	Sordariomycetes	Onygenaceae
	Cochliobolus	Blastocladiomycota	Obertrumia
	Nectriaceae	Atractiella	Galactomyces
	Galactomyces	RM2-SGM58	Hypocrea
	Blastocladiomycota	Dothideomycetes	Tilletiaria
		Trichosporon	Exobasidiomycetes
		Diaporthe	Fusarium
		Basidiomycota	
		Cystofilobasidiaceae	

**Enriched**	Pseudallescheria	Ascobolaceae	Pezizales
	Monosiga	Onygenaceae	Tremella
	Dissoconium	Nectriaceae	Talaromyces
	Glomeromycota	Pseudallescheria	Glomeromycota
	Craspedida	Pseudoperkinsidae	Nectriaceae
	Sordariales	Cochliobolus	Trichocomaceae
	Pezizales		Scytinostroma
	Agaricomycetes		Monosiga
	Cryptococcus		Pyxidiophora
	Sphaeroeca		Trichosporon
	Bryometopus		Eurotiomycetes
	Salpingoeca		Exobasidiales
	Atractiella		Cryptococcus
	Acrospermum		Craspedida
	Gibberella		Microbotryomycetes

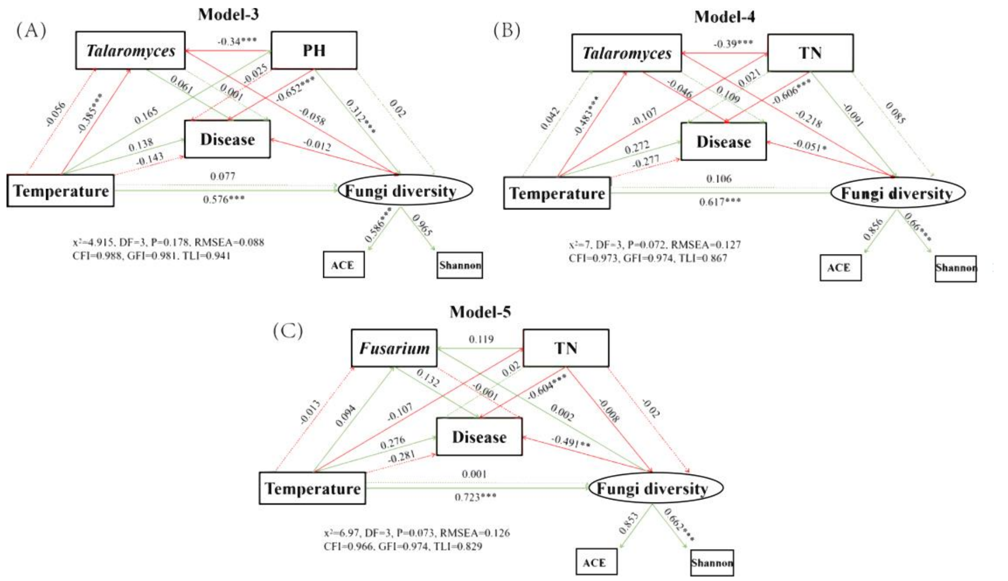

Figure S5. Structural Equation Modeling (SEM) analysis showed the contributions of *Talaromyces* (A and B), *Fusarium* (C), temperature, soil and fungi diversity to the sugarcane disease. Soil was represented by mainly compositions including pH and TN (Total Nitrogen); Fungi diversity included ACE and Shannon index. The numbers above the arrows indicate path coefficients. *p*-value: * < 0.05, ** < 0.01, *** < 0.001.

Table S2 Model parameters summary table

	X2	DF	P	CFI	GFI	TLI	RMR	RMSEA	AIC	BIC
Model-1	4.825	3	0.185	0.987	0.982	0.937	0.037	0.086	40.825	84.58
Model-2	1.282	3	0.733	1	0.995	1.054	0.018	0	37.282	81.037
Model-3	4.915	3	0.178	0.988	0.981	0.941	0.037	0.088	40.915	84.67
Model-4	7	3	0.072	0.973	0.974	0.867	0.049	0.127	43	86.755
Model-5	6.97	3	0.073	0.966	0.974	0.829	0.045	0.126	42.97	86.724

Table S3 Summary of model-1 internal path coefficients and normalized coefficients

Model-1	Unstandardized				Standardized				P
	Estimate	Total Effects	Direct Effects	Indirect Effects	Standardized Estimate	Standardized Total Effects	Standardized Direct Effects	Standardized Indirect Effects	
ungi diversity<-Temperature	0.58	0.63	0.58	0.05	0.601	0.653	0.601	0.052	***
Fungi diversity<-Fusarium	-0.074	-0.074	-0.074	0	-0.076	-0.076	-0.076	0	0.359
Shannon<-Fungi diversity	0.607	0.607	0.607	0	0.586	0.586	0.586	0	***
Fusarium<-Temperature	0.039	0.081	0.039	0.042	0.039	0.081	0.039	0.042	0.718
Disease<-Fungi diversity	0.021	0.021	0.021	0	0.02	0.02	0.02	0	0.871
Disease<-Temperature	0.085	-0.005	0.085	-0.09	0.085	-0.005	0.085	-0.09	0.43
Ace<-Fungi diversity	1	1	1	0	0.965	0.965	0.965	0	
Fungi diversity<-pH	0.338	0.32	0.338	-0.019	0.351	0.331	0.351	-0.02	***
Disease<-Fusarium	0.247	0.245	0.247	-0.002	0.247	0.245	0.247	-0.002	0.002
pH<-Temperature	0.165	0.165	0.165	0	0.165	0.165	0.165	0	0.127
Fusarium<-pH	0.256	0.256	0.256	0	0.256	0.256	0.256	0	0.017
Disease<-pH	-0.746	-0.677	-0.746	0.07	-0.746	-0.677	-0.746	0.07	***

Note: Specific values for each side of the model, including unstandardized and standardized data. *p*- value: * < 0.05, ** < 0.01, *** < 0.001. The same below.

Table S4 Summary of model-2 internal path coefficients and normalized coefficients

Model-2	Unstandardized				Standardized				P
	Estimate	Total Effects	Direct Effects	Indirect Effects	Standardized Estimate	Standardized Total Effects	Standardized Direct Effects	Standardized Indirect Effects	
Fungi diversity<-Temperature	0.615	0.631	0.615	0.016	0.676	0.693	0.676	0.017	***
Fungi diversity<-Fusarium	0.01	0.01	0.01	0	0.011	0.011	0.011	0	0.905
Shannon<-Fungi diversity	0.682	0.682	0.682	0	0.621	0.621	0.621	0	***
Disease<-Fungi diversity	-0.344	-0.344	-0.344	0	-0.313	-0.313	-0.313	0	0.014
Fusarium<-Temperature	0.089	0.081	0.089	-0.008	0.089	0.081	0.089	-0.008	0.422
Disease<-Temperature	0.354	-0.005	0.354	-0.359	0.354	-0.005	0.354	-0.359	***
Fungi diversity<-AN	0.082	0.082	0.082	0	0.09	0.09	0.09	0	0.333
Ace<-Fungi diversity	1	1	1	0	0.91	0.91	0.91	0	
AN<-Temperature	0.184	0.184	0.184	0	0.184	0.184	0.184	0	0.088
Disease<-Fusarium	0.029	0.025	0.029	-0.003	0.029	0.025	0.029	-0.003	0.653
Fusarium<-AN	-0.044	-0.044	-0.044	0	-0.044	-0.044	-0.044	0	0.69
Disease<-AN	-0.781	-0.81	-0.781	-0.029	-0.781	-0.81	-0.781	-0.029	***

Note: Specific values for each side of the model, including unstandardized and standardized data. *p*- value: * < 0.05, ** < 0.01, *** < 0.001. The same below.

Table S5 Summary of model-3 internal path coefficients and normalized coefficients

Model-3	Unstandardized				Standardized				P
	Estimate	Total Effects	Direct Effects	Indirect Effects	Standardized Estimate	Standardized Total Effects	Standardized Direct Effects	Standardized Indirect Effects	
Fungi diversity<-Temperature	0.556	0.63	0.556	0.074	0.576	0.653	0.576	0.077	***
Fungi diversity<-Talaromyces	-0.056	-0.056	-0.056	0	-0.058	-0.058	-0.058	0	0.547
Talaromyces<-Temperature	-0.385	-0.441	-0.385	-0.056	-0.385	-0.441	-0.385	-0.056	***
Shannon<-Fungi diversity	0.607	0.607	0.607	0	0.586	0.586	0.586	0	***
Disease<-Fungi diversity	-0.013	-0.013	-0.013	0	-0.012	-0.012	-0.012	0	0.925
Disease<-Temperature	0.138	-0.005	0.138	-0.143	0.138	-0.005	0.138	-0.143	0.24
Disease<-Talaromyces	0.061	0.062	0.061	0.001	0.061	0.062	0.061	0.001	0.533
Ace<-Fungi diversity	1	1	1	0	0.965	0.965	0.965	0	
Fungi diversity<-pH	0.301	0.32	0.301	0.019	0.312	0.331	0.312	0.02	***
pH<-Temperature	0.165	0.165	0.165	0	0.165	0.165	0.165	0	0.127
Talaromyces<-pH	-0.34	-0.34	-0.34	0	-0.34	-0.34	-0.34	0	***
Disease<-pH	-0.652	-0.677	-0.652	-0.025	-0.652	-0.677	-0.652	-0.025	***

Note: Specific values for each side of the model, including unstandardized and standardized data. *p*- value: * < 0.05, ** < 0.01, *** < 0.001. The same below.

Table S6 Summary of model-4 internal path coefficients and normalized coefficients

Model-4	Unstandardized				Standardized				P
	Estimate	Total Effects	Direct Effects	Indirect Effects	Standardized Estimate	Standardized Total Effects	Standardized Direct Effects	Standardized Indirect Effects	
Fungi diversity<-Temperature	0.529	0.619	0.529	0.091	0.617	0.723	0.617	0.106	***
Fungi diversity<-Talaromyces	-0.186	-0.186	-0.186	0	-0.218	-0.218	-0.218	0	0.061
Talaromyces<-Temperature	-0.483	-0.441	-0.483	0.042	-0.483	-0.441	-0.483	0.042	***
Shannon<-Fungi diversity	0.771	0.771	0.771	0	0.66	0.66	0.66	0	***
Disease<-Fungi diversity	-0.585	-0.585	-0.585	0	-0.501	-0.501	-0.501	0	0.012

Disease<-Temperature	0.272	-0.005	0.272	-0.277	0.272	-0.005	0.272	-0.277	0.083
Disease<-Talaromyces	-0.046	0.063	-0.046	0.109	-0.046	0.063	-0.046	0.109	0.688
Ace<-Fungi diversity	1	1	1	0	0.856	0.856	0.856	0	
Fungi diversity<-TN	-0.078	-0.005	-0.078	0.073	-0.091	-0.006	-0.091	0.085	0.386
TN<-Temperature	-0.107	-0.107	-0.107	0	-0.107	-0.107	-0.107	0	0.327
Talaromyces<-TN	-0.39	-0.39	-0.39	0	-0.39	-0.39	-0.39	0	***
Disease<-TN	-0.606	-0.585	-0.606	0.021	-0.606	-0.585	-0.606	0.021	***

Note: Specific values for each side of the model, including unstandardized and standardized data. *p*-value: * < 0.05, ** < 0.01, *** < 0.001. The same below.

Table S7 Summary of model-5 internal path coefficients and normalized coefficients

Model-5	Unstandardized				Standardized				P
	Estimate	Total Effects	Direct Effects	Indirect Effects	Standardized Estimate	Standardized Total Effects	Standardized Direct Effects	Standardized Indirect Effects	
Fungi diversity<-Temperature	0.617	0.618	0.617	0.001	0.723	0.724	0.723	0.001	***
Fungi diversity<-Fusarium	0.001	0.001	0.001	0	0.002	0.002	0.002	0	0.987
Shannon<-Fungi diversity	0.776	0.776	0.776	0	0.662	0.662	0.662	0	***
Disease<-Fungi diversity	-0.576	-0.576	-0.576	0	-0.491	-0.491	-0.491	0	0.009
Fusarium<-Temperature	0.094	0.081	0.094	-0.013	0.094	0.081	0.094	-0.013	0.391
Disease<-Temperature	0.276	-0.005	0.276	-0.281	0.276	-0.005	0.276	-0.281	0.081
Ace<-Fungi diversity	1	1	1	0	0.853	0.853	0.853	0	
Fungi diversity<-TN	-0.007	-0.007	-0.007	0	-0.008	-0.008	-0.008	0	0.934
Disease<-Fusarium	0.132	0.131	0.132	-0.001	0.132	0.131	0.132	-0.001	0.125
TN<-Temperature	-0.107	-0.107	-0.107	0	-0.107	-0.107	-0.107	0	0.327
Fusarium<-TN	0.119	0.119	0.119	0	0.119	0.119	0.119	0	0.277
Disease<-TN	-0.604	-0.585	-0.604	0.02	-0.604	-0.585	-0.604	0.02	***

Note: Specific values for each side of the model, including unstandardized and standardized data. *p*-value: * < 0.05, ** < 0.01, *** < 0.001.

Table S8. Summary of mean NTI and βNTI during fungal assembly.

	averageNTI	βNTI	Contribution ratio of random factors
Seeding	3.94	0.38	5.36
Tillering	4.66	-0.28	9.50
Elongation	4.17	0.72	16.50
Maturity	3.95	0.51	4.38
Seeding-Tillering		-0.38	3.59
Tillering-Elongation		0.51	8.59
Elongation-Maturity		0.39	2.74

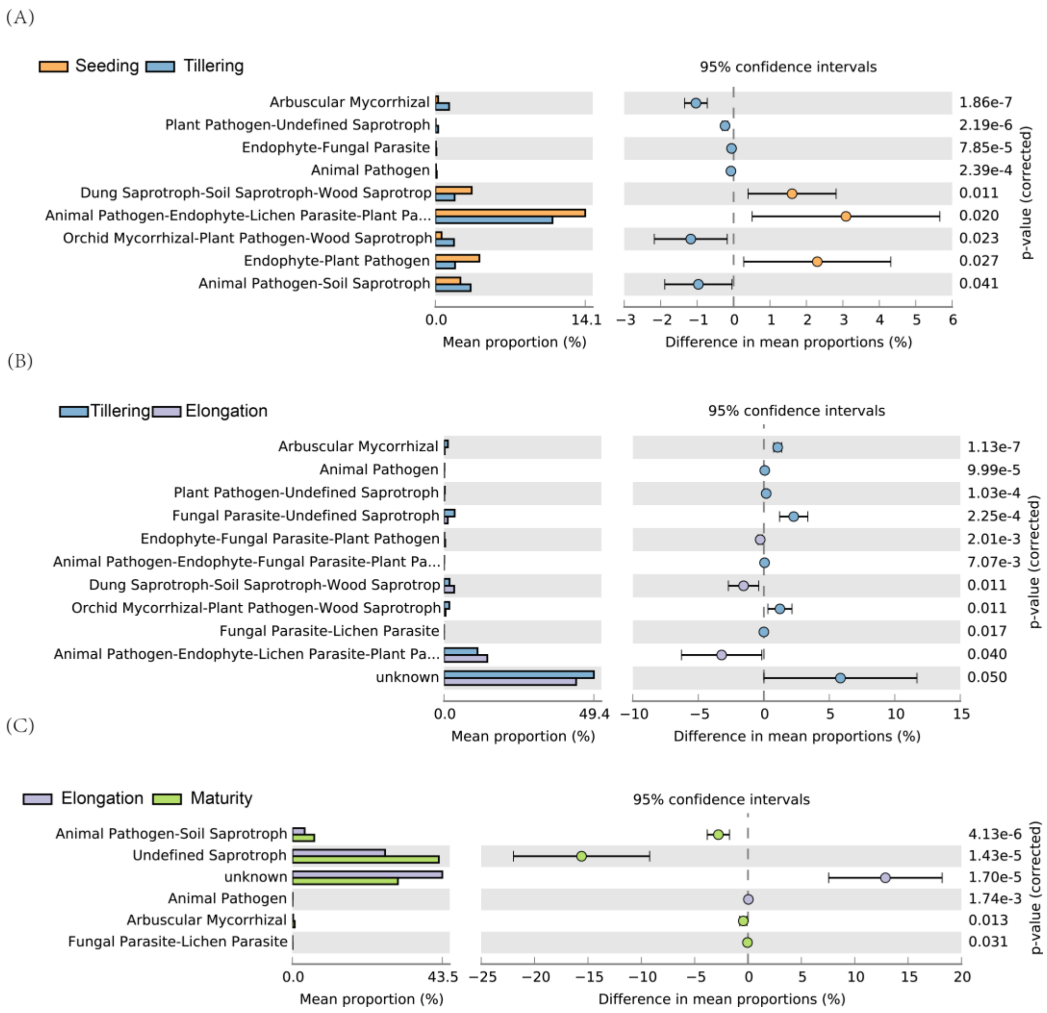

Figure S6. The functional classification with significant functional difference phases in different seasons was obtained based on FUNGuild database, differences in seeding, tillering, elongation and maturity (orange, blue, purple and green strips, respectively).

Table S9 Network structure parameters summary table

	total positive	total negative	average density	mean degree	closeness
Seeding	504	366	0.28	8.03	29.00

Tillering	430	436	0.23	6.65	29.13
Elongation	476	382	0.21	5.94	29.07
Maturity	452	418	0.25	7.39	29.00

Note: Co-occurrence network based on spearman correlation (Top 30 fungal genera, $|r| > 0.1$, $p < 0.05$)
